# P-1276. Remdesivir and Obeldesivir Retain Potent Activity Against SARS-CoV-2 Omicron Variants

**DOI:** 10.1093/ofid/ofaf695.1466

**Published:** 2026-01-11

**Authors:** Lauren Rodriguez, J Lizbeth Reyes Zamora, Jiani Li, Dong Han, Nadine Peinovich, Clarissa Martinez, Pui Yan Ho, Ross Martin, John P Bilello, Jason K Perry, Charlotte Hedskog

**Affiliations:** Gilead Sciences, Inc., Foster City, CA; Gilead Sciences, Inc., Foster City, CA; Gilead Sciences, Inc., Foster City, CA; Gilead Sciences, Inc., Foster City, CA; Gilead Sciences, Inc., Foster City, CA; Gilead Sciences, Inc., Foster City, CA; Gilead Sciences, Inc., Foster City, CA; Gilead Sciences, Foster City, California; Gilead Sciences, Inc., Foster City, CA; Gilead Sciences, Inc., Foster City, CA; Gilead Sciences, Inc., Foster City, CA

## Abstract

**Background:**

Remdesivir (RDV), a nucleotide analog prodrug targeting the SARS-CoV-2 RNA-dependent RNA polymerase (Nsp12), is approved to treat COVID-19 in hospitalized and nonhospitalized patients. Obeldesivir (ODV), an oral mono-5’-isobutyryl ester nucleoside prodrug, is metabolized into the same active triphosphate as RDV. RDV and ODV retained antiviral activity against previous Omicron subvariants (BA.1 to JN.1) relative to the ancestral WA1 strain. This study evaluated RDV and ODV antiviral activity against recent Omicron subvariants (XBB.2, BA.2.86.1, JN.1.7, KP.2, KP.3.3, KP.3.1.1, LP.8.1 and XEC) using clinical isolates and Nsp12-mutant replicons.

**Methods:**

Nsp12 substitutions in Omicron subvariants were identified using GISAID EpiCoV sequence database and structural effects were analyzed via a cryo-EM–based model of the replication-transcription complex. Antiviral activity (half-maximal effective concentration [EC_50_]) of RDV and ODV against subvariant clinical isolates was assessed by nucleoprotein ELISA in A549-hACE2-TMPRSS2 cells and by site-directed mutants in a replicon system.

**Results:**

Among >16 million SARS-CoV-2 sequences, only 5 Nsp12 lineage-defining substitutions (in ≥75% of sequences: D63N, Y273H, P323L, G671S, G823insD) were identified since the beginning of the pandemic. No new defining substitutions in Nsp12 were identified in XBB.2, BA.2.86.1, JN.1.7, KP.2, KP.3.3, KP.3.1.1, LP.8.1, or XEC compared with earlier Omicron subvariants. Phenotyping of clinical isolates of XBB.2, BA.2.86.1, JN.1.7, KP.2, KP.3.3, KP.3.1.1, and XEC indicated no change of RDV or ODV in vitro antiviral activity (≤0.86-fold change). Phenotyping of a replicon of LP.8.1 showed no loss of RDV or ODV susceptibility (≤1.14-fold change). Lineage-defining substitutions in other regions of the replication complex (Nsp8, Nsp9, Nsp10, Nsp13 and Nsp14) have been observed in the clinical isolates tested. The presence of these substitutions did not impact the susceptibility to RDV and ODV.

**Conclusion:**

RDV and ODV retained in vitro antiviral activity against all tested Omicron subvariants with potencies comparable to reference strains.

**Disclosures:**

Lauren Rodriguez, PhD, Gilead Sciences, Inc.: Employee|Gilead Sciences, Inc.: Stocks/Bonds (Public Company) J. Lizbeth Reyes Zamora, PhD, Gilead Sciences, Inc.: Employee|Gilead Sciences, Inc.: Stocks/Bonds (Public Company) Jiani Li, PhD, Gilead Sciences, Inc.: Employee|Gilead Sciences, Inc.: Stocks/Bonds (Public Company) Dong Han, MS, Gilead Sciences, Inc.: Employee|Gilead Sciences, Inc.: Stocks/Bonds (Public Company) Nadine Peinovich, MPH, Gilead Sciences, Inc.: Employee|Gilead Sciences, Inc.: Stocks/Bonds (Public Company) Clarissa Martinez, MPH, Gilead Sciences, Inc.: Employee|Gilead Sciences, Inc.: Stocks/Bonds (Public Company) Pui Yan Ho, PhD, Gilead Sciences, Inc.: Employee|Gilead Sciences, Inc.: Stocks/Bonds (Public Company) Ross Martin, PhD, Gilead Sciences, Inc.: Employee|Gilead Sciences, Inc.: Stocks/Bonds (Public Company) John P. Bilello, PhD, Gilead Sciences, Inc.: Employee|Gilead Sciences, Inc.: Stocks/Bonds (Public Company) Jason K. Perry, PhD, Gilead Sciences, Inc.: Employee|Gilead Sciences, Inc.: Stocks/Bonds (Public Company) Charlotte Hedskog, PhD, Gilead Sciences, Inc.: Employee|Gilead Sciences, Inc.: Stocks/Bonds (Public Company)

